# Antibody titer after administration of mRNA‐based vaccine against severe acute respiratory syndrome coronavirus 2 in liver transplant recipients

**DOI:** 10.1002/ags3.12677

**Published:** 2023-04-19

**Authors:** Atsuyoshi Mita, Yasunari Ohno, Yuichi Masuda, Kazuki Yoshizawa, Koji Kubota, Tsuyoshi Notake, Akira Shimizu, Hidetoshi Matsunami, Yuji Soejima

**Affiliations:** ^1^ Division of Gastroenterological, Hepato‐Biliary‐Pancreatic, Transplantation and Pediatric Surgery, Department of Surgery Shinshu University School of Medicine Matsumoto Japan; ^2^ Matsunami General Hospital Gifu Japan

**Keywords:** liver transplant, mRNA‐based vaccine, positive humoral response, severe acute respiratory syndrome coronavirus 2, vaccination‐induced portal vein thrombosis

## Abstract

**Introduction:**

The mRNA‐based vaccine was released as a COVID‐19 prophylactic; however, its efficacy in organ transplant recipients is unknown. This study aimed to clarify this in liver transplant recipients.

**Methods:**

Herein, liver transplant recipients from two hospitals who received vaccines were included. Immunoglobulin‐G antibodies against the spike and nucleocapsid proteins were measured chronologically after the second, third, and fourth vaccine doses.

**Results:**

Antibody levels in 125 liver transplant recipients and 20 healthy volunteers were analyzed. The median age at transplant was 35 (interquartile range 1, 53) years, and the period between transplant and the first dose was 15.2 ± 7.7 years. After the second and third doses, 89.1% and 100% of recipients displayed a positive humoral response, respectively. Anti‐spike antibodies after the second dose were significantly reduced at 3 and 6 months, compared to that at 1 month (26.0 [5.4, 59.5], 14.7 [6.5, 31.4] vs. 59.7 [18.3, 164.0] AU/mL, respectively, *p* < 0.0001). However, a booster vaccine significantly elevated anti‐spike antibodies in LT recipients (*p* < 0.0001) as well as in healthy controls (*p* < 0.0001). Additionally, the decay rate was comparable between the transplant recipients and controls (2.1 [0.8, 4.5] vs. 2.7 [1.1, 4.1] AU/mL/day, *p* = 0.9359). Only 4.0% of vaccinated transplant recipients were positive for anti‐nucleocapsid antibodies.

**Conclusion:**

Liver transplant recipients can acquire immunity similar to that of healthy people through vaccination against SARS‐CoV‐2. The antibody decay rate is the same, and booster vaccinations should be administered similarly to that in healthy individuals.

## Graphical abstract

After the second and third doses of mRNA‐based vaccine against SARS‐CoV‐2, 89.1% and 100% of liver transplant recipients displayed a positive humoral response, respectively. A booster vaccine significantly elevated anti‐spike antibodies in liver transplant recipients.

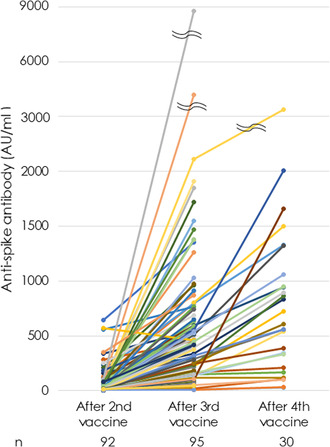

## 1 INTRODUCTION

The new coronavirus disease 2019 (COVID‐19) caused a pandemic in 2019. An mRNA‐based vaccine against severe acute respiratory syndrome coronavirus 2 (SARS‐CoV‐2) was rapidly released for prophylaxis, and its high efficacy has been reported.^1^ The vaccine reportedly had insufficient efficacy in preventing COVID‐19 in organ transplant recipients.^2^, ^3^, ^4^, ^5^ The Pfizer‐BioNTech BNT162b2 mRNA‐based vaccine elicited more inferior humoral immunity in liver transplant (LT) recipients than that in healthy volunteers,^3^ and induced lower specific T cell responses in solid organ transplant recipients than that in immunocompetent subjects.^5^ Nevertheless, many countries in Europe, the United States, and Japan recommend vaccination in LT recipients.^6^, ^7^ However, the vaccine efficacy is still unknown in organ transplant recipients.

In addition to mRNA‐based vaccines, several types of vaccines, such as viral vector‐based vaccines, have recently become available. The Pfizer‐BioNTech BNT162b2 was initially developed based on a nucleoside‐modified mRNA vector vaccine encoding the pre‐fusion spike glycoprotein of SARS‐CoV‐2 and approved worldwide.^1^ The mRNA‐based vaccines have been reported to have higher efficacy compared to other vaccines^8^ and are now more commonly used in Japan.

SARS‐CoV‐2 has four key structural proteins: the spike (S‐), matrix, envelope, and nucleocapsid (N‐) proteins.^9^ Immunoglobulin‐G (IgG), developed against the S‐protein, is believed to be a neutralizing antibody (Ab) immune response and is currently the primary target for SARS‐CoV‐2 vaccine trials. In many previous studies, the humoral response of the vaccine was evaluated by measuring Ab titers against the S‐protein, instead of following up on subsequent infection rates.^3,4^ On the other hand, IgG developed against the N‐protein is thought to reflect past SARS‐CoV‐2 infections.^9^

Here, we aimed to clarify the efficacy of SARS‐CoV‐2 mRNA‐based vaccines by measuring serum Ab titers against the S‐ and N‐proteins after vaccination in LT recipients.

## 2 PATIENTS AND METHODS

This study was designed as a prospective cohort from two Japanese hospitals (Shinshu University Hospital in Matsumoto and Matsunami General Hospital in Gifu). The inclusion criteria were LT recipients who had received two or more doses of an mRNA‐based vaccine against SARS‐CoV‐2. A SARS‐CoV‐2 infection history before vaccination was not considered, and patients who received the vaccine before LT were excluded. Patient data, including age at LT and first vaccination, sex, type of donor and graft, relationship to donor, body mass index, immunosuppression regimen at first vaccination, duration between LT and vaccinations, and history of SARS‐CoV‐2 infection, were collected from their medical records and a questionnaire.

IgG Ab titers against the S‐ and N‐proteins were measured at 1, 3, 6, 9, and 12 months after the second dose and at 1, 3, and 6 months after the third dose through outpatient visits to the hospitals. Abs levels were also measured 1 month before and 1 and 6 months after the third dose in 20 healthy volunteers (medical staff) who received the vaccine as controls.

Collected blood samples were immediately separated into serum by centrifugation at 3500 rotations per minute for 6 min and stored at −20°C for 1 to 2 weeks. The samples were sent to a laboratory (SRL Inc.) where SARS‐CoV‐2 antibodies against the S‐ and N‐proteins were measured by chemiluminescent enzyme immunoassay using SARS‐CoV‐2 S‐IgG measurement reagent (FUJIREBIO) and electrochemiluminescence immunoassay using Elecsys Anti‐SARS‐CoV‐2 (Rosche Diagnostics K.K.), respectively. Titers <1.0 arbitrary units (AU)/mL were considered negative according to the manufacturer's instructions.

### 2.1 Vaccination

The mRNA‐based vaccines (Pfizer‐BioNTech BNT162b2 or Moderna mRNA‐1273) were administered according to Japanese government protocol: 3‐week intervals between the first and second doses, followed by the third dose at more than 6 months. We recommended that LT recipients take the first dose of the vaccine more than 6 months after the operation.

### 2.2 Calculating decay rates

Based on a previous report,^10^ the decay rate of Ab titers was calculated by dividing the difference between maximum and minimum titers by interval days in two points after the third and before the fourth vaccine dose.

### 2.3 Ethics declarations

The study was conducted in accordance with the Declaration of Helsinki, and the protocol was approved by the Ethics Committee of Shinshu University (registration number: 5265). Written informed consent was obtained from all the patients and/or their families before inclusion.

### 2.4 Statistical analyses

Continuous variables are presented as mean ± standard deviation (SD) for normally distributed data or as median (interquartile range) for non‐normally distributed data. Categorical variables are presented as proportions. Patient characteristics were compared using the Student's *t*‐test or Mann–Whitney *U* test for continuous variables and the chi‐squared or Fisher's exact tests for categorical variables. The change in Ab titers in each patient was tested using a paired *t*‐test. The correlation between Ab titers and interval days after vaccination was evaluated using Pearson's product–moment correlation coefficient.

A logistic regression analysis identified the individual predictors of a positive humoral response. Factors associated with Ab positivity in the univariate analysis were included in the multivariate analysis. Continuous variables were transformed into categorical variables through cutoff values analyzed using receiver operating characteristic curve analysis for patient age at LT.

All statistical tests were two‐sided and performed using JMP software, version 16 (SAS Institute); statistical significance was set at *p* < 0.05.

## 3 RESULTS

Out of the initial 126 LT recipients enrolled in this study, one was excluded due to having the first vaccination before the transplant. The reasons for the LT varied, with 64, 49, 11, and 1 being due to biliary, hepatocellular disease, metabolic liver disease, and multiple liver cysts, respectively. The median age at LT was 35 (interquartile range 1, 53) years, with 91.2% receiving the organ from a living donor. The first vaccine dose was taken at 15.2 ± 7.7 years after LT, at a median age of 48 (interquartile range 25, 67) years, with 96% of LT recipients receiving the Pfizer‐BioNTech BNT162b2 (Table 1). At the time of the first vaccination, the post‐transplant immunosuppression regimen included calcineurin inhibitors (*n* = 122, 97.6%), antimetabolites such as mycophenolate mofetil (MMF) and azathioprine (*n* = 40, 32.0%), steroids (*n* = 22, 17.6%), and mammalian target of rapamycin inhibitors (*n* = 6, 4.8%). The number of immunosuppressants administered varied between a single reagent (*n* = 77), two reagents (*n* = 33), three reagents (*n* = 14), and four reagents (*n* = 1).

**TABLE 1:** Patient characteristics in liver transplant recipients.

Variable	*n* = 125
Age@LT, years old [median (IQR)]	35 (1, 53)
Age@Vac, years old [median (IQR)]	48 (25, 67)
Duration between LT & Vac	15.2 ± 7.7
Female, *n* (%)	67 (53.6)
Primary disease, *n* (%)	
Biliary disease	64 (51.2)
Hepatocellular disease	49 (39.2)
Metabolic liver disease	11 (8.8)
Others	1 (0.8)
LDLT, *n* (%)	114 (91.2)
Donor, *n* (%)	
Parents	54 (43.2)
Children	31 (24.8)
Non‐kinship	21 (16.8)
Others	19 (15.2)
Immunosuppression, *n* (%)	
CNI	122 (97.6)
Antimetabolite	40 (32.0)
Steroid	22 (17.6)

Abbreviations: CNI, calcineurin inhibitor; LDLT, living donor liver transplantation; LT, liver transplant; Vac, vaccination of SARS‐CoV‐19.

**FIGURE 1:**
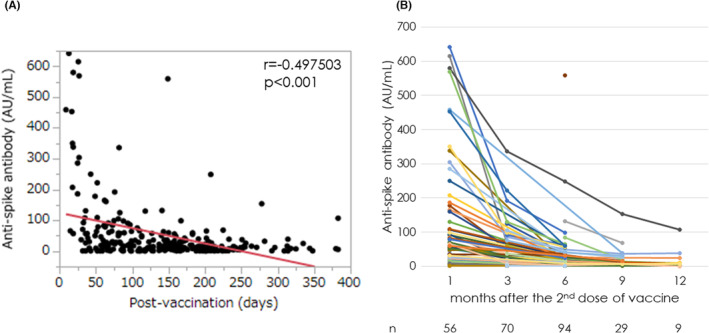
Anti‐spike protein antibody (anti‐S Ab) titers are plotted for 116 liver transplant (LT) recipients whose Abs were measured after the second dose and until the third dose of the mRNA‐based vaccine against severe acute respiratory syndrome coronavirus 2 (SARS‐CoV‐2). 1a: Scatterplot correlation between Ab titer and interval days after the second vaccine dose. 1b: Ab titers are plotted 1, 3, 6, 9, and 12 months after the second dose and until the third dose for each LT recipient. The number of samples at each time point is shown below the graph. Ab titers decreased over time in most recipients (*n* = 106, 91.4%) although titer values varied.

Among the 258 samples from 116 LT recipients, an inverse correlation was noted between anti‐S Ab titers and interval days after the second dose (*r* = −0.497503, *p* < 0.001, Figure 1A). Ab titers were plotted for each LT recipient at 1, 3, 6, 9, and 12 months between the second and third doses, and were found to decrease over time in most cases (*n* = 106, 91.4%, Figure 1B). The median anti‐S Abs reached 59.7 (18.3, 164.0) AU/mL 1 month after the second dose, followed by a significant decrease to 26.0 (5.4, 59.5), 14.7 (6.5, 31.5), and 11.7 (7.2, 17.0) AU/mL at 3, 6, and 9 months after the second dose, respectively, compared to those at 1 month after (*p* < 0.0001). Abs reached a positive humoral response (≥1.0 Au/mL) in 89.1% of recipients whose anti‐S Abs could be measured after the second dose and before the third dose. This anti‐S Ab positive response was maintained in 86.5% of recipients just before the third dose.

Factors influencing a positive humoral response to anti‐S Abs after the second dose were explored by regression analysis. Age less than 47 years at LT, a duration of more than 2 years between LT and the first vaccination, and a single immunosuppression regimen were significantly associated with the positivity of anti‐S Abs by univariate analysis. Among these factors, an age <47 years at LT and a duration >2 years between LT and the first vaccination were independent predictors of anti‐S Ab positivity by multivariate analysis (Table 2).

**TABLE 2:** Regression analysis for predicting positive humoral response after the second vaccine dose in liver transplant recipients.

Variable	Univariate analysis		Multivariate analysis	
	OR	*p*‐Value	OR	*p*‐Value
Age@LT <47 years	11.5 (2.4, 54.7)	0.0002	9.3 (1.5, 55.9)	0.0154
Duration between LT‐Vac >2 years	12.4 (3.4, 45.1)	0.0002	8.1 (1.6, 40.5)	0.0108
BMI	1.2 (0.95, 1.42)	0.1175		
Relationship with donor, consanguinity	2.5 (0.7, 9.0)	0.1898		
Single immunosuppression	23.0 (2.9, 184.0)	0.0031	7.0 (0.8, 64.9)	0.0837
Antimetabolite	0.5 (0.1, 1.5)	0.2016		
Steroid	0.7 (0.2, 2.8)	0.5994		

Abbreviations: BMI, Body mass index; LT, liver transplant; OR, odds ratio; Vac, vaccination of SARS‐CoV‐19.

**FIGURE 2:**
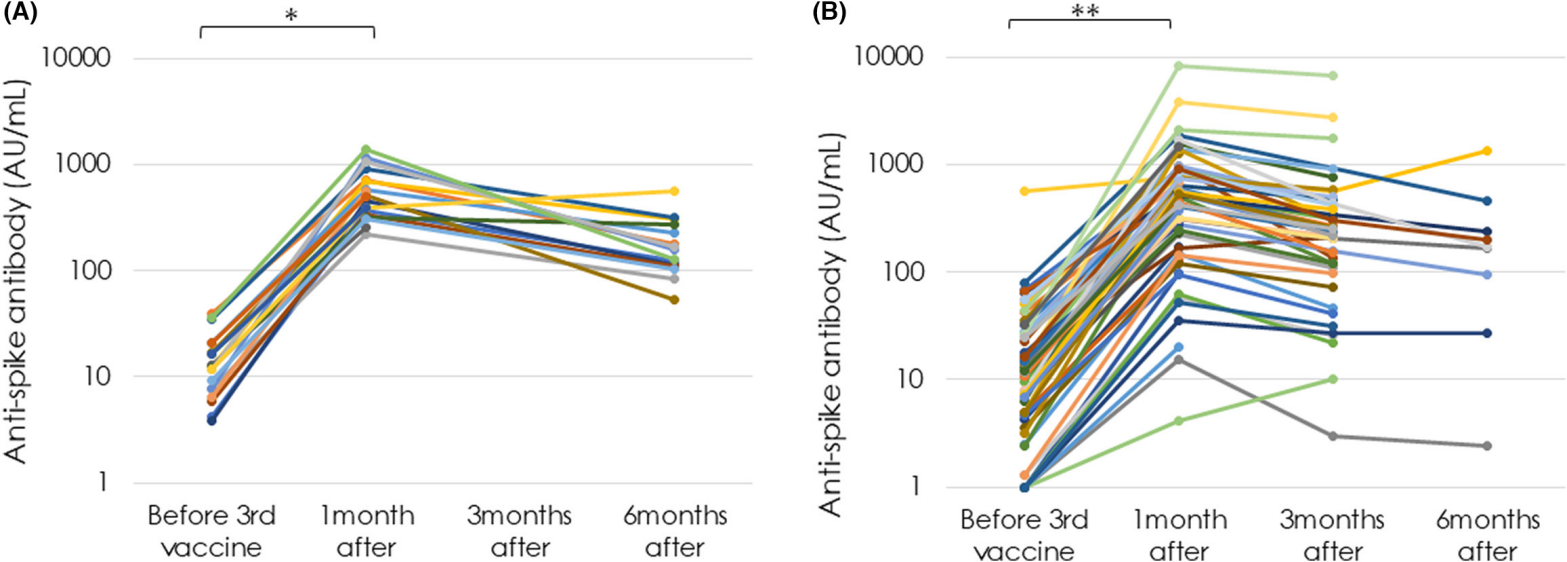
Anti‐spike protein antibody (anti‐S Ab) titers are plotted for 20 healthy controls (A) and 89 liver transplant (LT) recipients (B) who took three doses of the mRNA‐based vaccine. Graphs show a logarithm Y axis because some LT recipients had very high Ab titer levels. Anti‐S Abs that were reduced before the third dose, significantly increased one month after the third dose (**p < 0.0001, B) as well as in 20 controls (*p < 0.0001, A), as determined using the paired *t*‐test. Anti‐S Abs decreased over time after the third dose in both healthy controls and LT recipients.

Ninety‐three LT recipients whose Ab titers were measured before and after the third dose had comparable ages (46.1 ± 21.1 vs. 42.5 ± 9.2, *p* = 0.4581) and body mass index (22.2 ± 3.3 vs. 23.2 ± 3.5, *p* = 0.2978), but the number of females was among them was more (53.8% vs. 20.0%, *p* = 0.0046) and the duration between the second and third doses of vaccine was shorter (46.1 ± 21.1 vs. 42.5 ± 9.2, *p* = 0.4581), compared with that in 20 healthy controls (Table 3). Anti‐S Abs that were reduced six or more months after the second dose significantly increased 1 month after the third dose in the controls (*n* = 20; *p* < 0.0001, Figure 2A) as well as in 93 LT recipients (*p* < 0.0001, Figure 2B). In addition, the median titers were comparable between these recipients and the controls, both before and after the third vaccination (12.0 [4.9, 23.6] vs. 12.2 [7.7, 20.0] AU/mL, *p* = 0.6300 and 438.0 [176.0, 767.5] vs. 505.5 [340.8, 710.8] AU/mL, *p* = 0.2076, respectively).

**TABLE 3:** Patient characteristics in healthy controls and liver transplant recipients who took the third vaccine dose.

Variables	Control, *n* = 20	LT recipient, *n* = 93	*p*‐Value
Age@Vac, years old (average)	42.5 ± 9.2	46.1 ± 21.1	0.4581
Female, *n* (%)	4 (20.0)	50 (53.8)	0.0046
BMI (average)	23.2 ± 3.5	22.2 ± 3.3	0.2978
Duration between second and third dose, months (average)	9.0 ± 0.3	7.6 ± 1.3	<0.0001

Abbreviations: BMI, Body mass index; Vac, vaccination of SARS‐CoV‐19.

Anti‐S Abs also decreased over time after the second and third doses (Figure 2B). When the decay rate was calculated by dividing the difference between maximum and minimum titers by interval days at two points after the third and before the fourth dose, the rate was similar between LT recipients and healthy controls (2.1 [0.8, 4.5] vs. 2.7 [1.1, 4.1] AU/mL/day, *p* = 0.9359).

**FIGURE 3:**
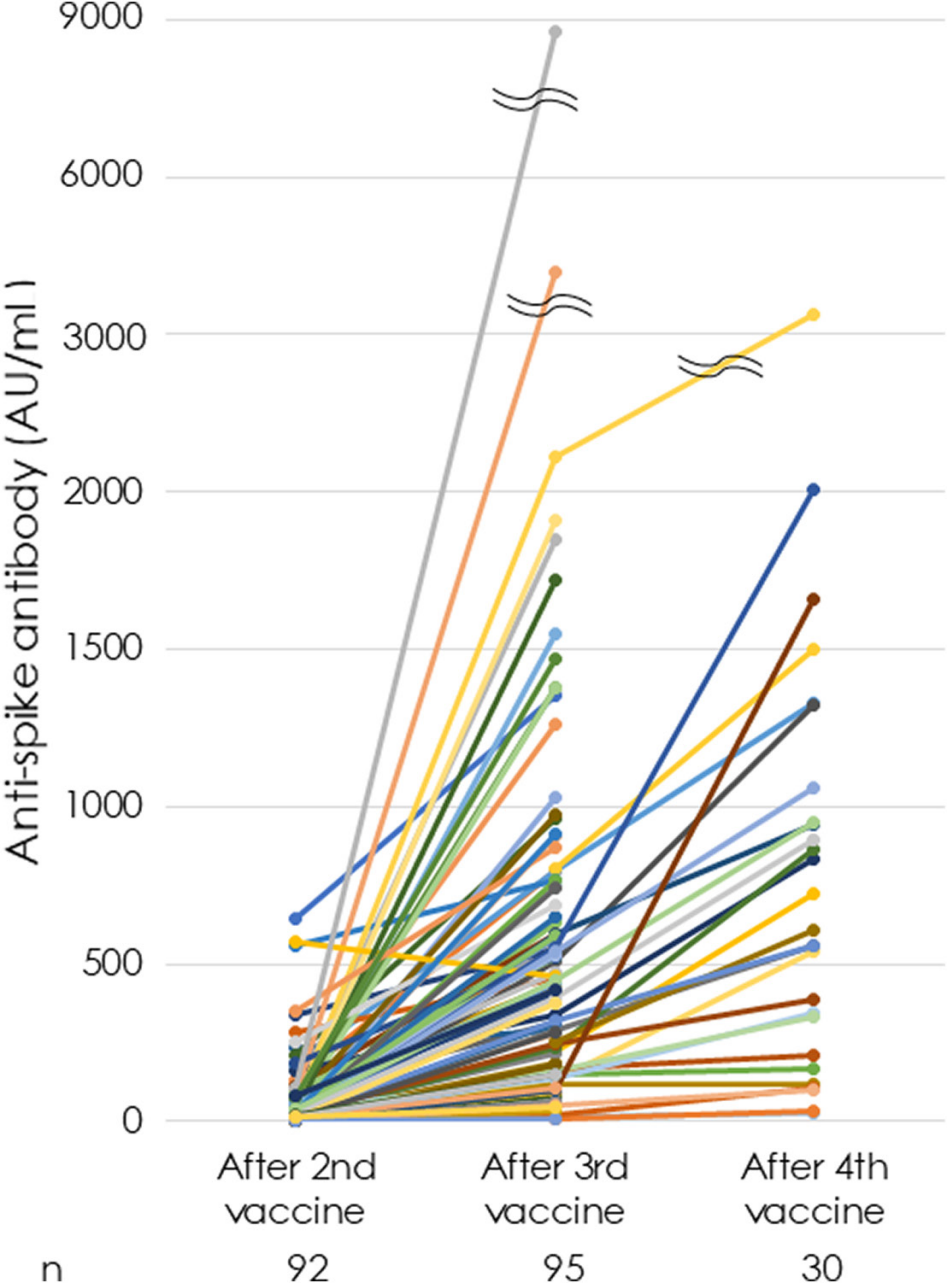
The peak anti‐spike protein antibody (anti‐S Ab) titers after the second, third, and fourth vaccine doses are plotted for 95 out of 125 liver transplant (LT) recipients whose Ab titers were measured at more than two points. The number of cases at each time point is shown below the graph. Abs levels were increased sequentially after each dose.

Anti‐S Abs were positive in 100% of the 93 LT recipients after the third dose. The peak Ab titer was widely different in each patient, ranging from a minimum of 1 to a maximum of 8600 AU/mL (Figure 2B). Peak titers plotted after the second, third, and fourth doses in 95 LT recipients revealed they elevated incrementally in 97.9% of recipients, indicating that multiple vaccine doses provide a boosting effect (Figure 3). Median peak anti‐S Abs increased significantly by 26.9 (9.8, 77.9), 417.0 (148.0, 743.0), and 557.5 (170.0, 977.5) AU/mL after the second, third, and fourth doses, respectively (*p* < 0.0001).

Anti‐nucleocapsid (Anti‐N) Ab was positive in only five LT recipients (4.0%), two of whom were already positive at the first Ab titer (21.8 and 5.6 AU/mL, respectively) because of pre‐vaccination infection. The remaining three tested positive because of either infection after two doses or asymptomatic infection after three doses. All the patients recovered fully without any prognostic symptoms after infection.

**FIGURE 4:**
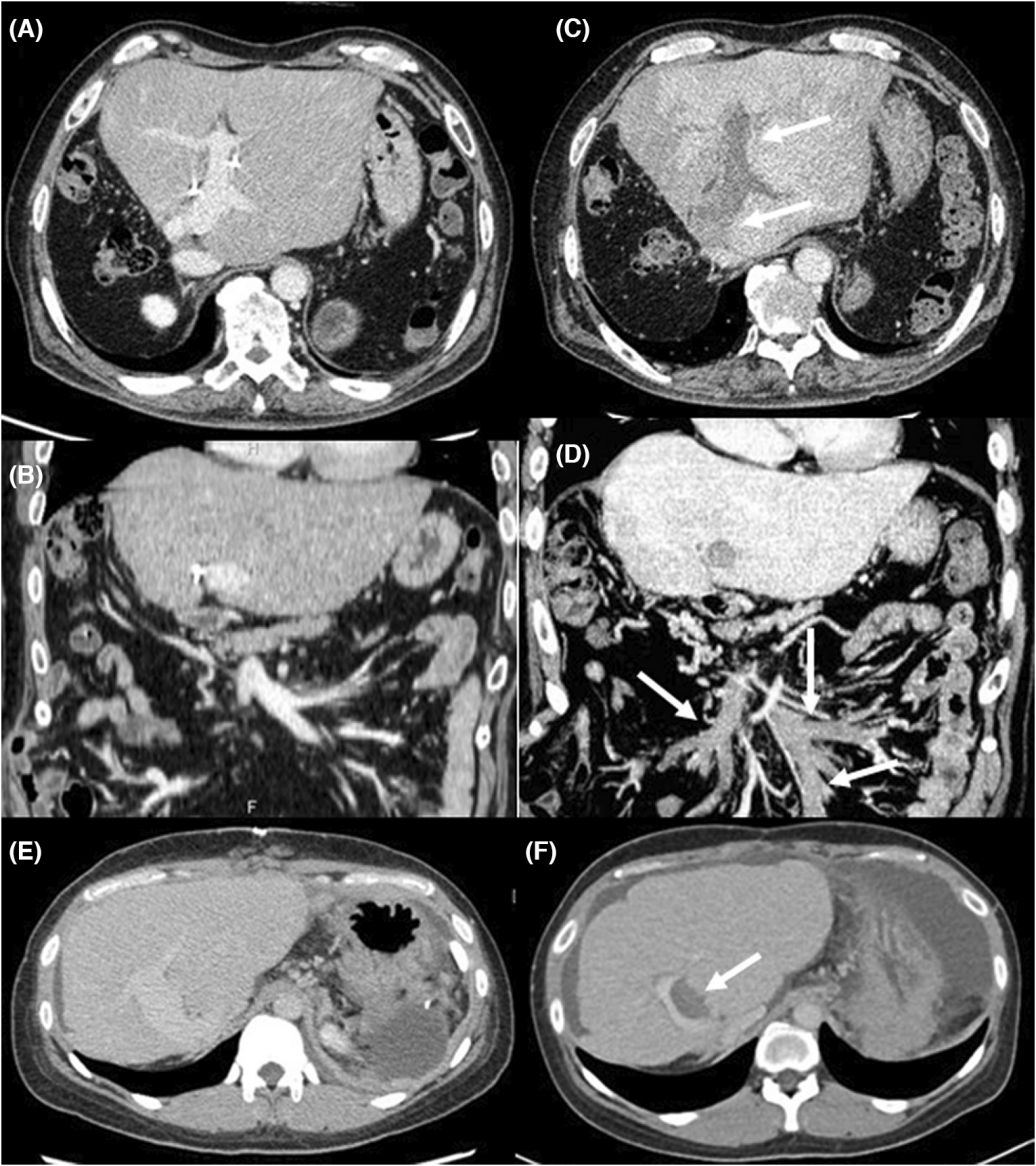
Enhanced abdominal computed tomography. Case 1 (A–D) and Case 2 (E,F). The portal vein was patent 1 year before the third vaccine dose in Case 1 (A and B) and before the fourth dose in Case 2 (E). However, the portal and superior mesenteric veins were completely obstructed by thrombosis 1 month after the third dose in Case 1 (C and D, arrows). Partial thrombosis in the portal vein was observed 2 weeks after the fourth dose in Case 2 (F, arrows).

Most LT recipients experienced only mild adverse effects such as fatigue, local pain, and fever after vaccination; however, two patients developed portal vein thrombosis. Case 1 was a 58‐year‐old male who had undergone a living donor LT with splenectomy for decompensated liver cirrhosis due to non‐alcoholic steatohepatitis using a left hemi‐liver graft 4 years earlier. A direct oral anticoagulant was administered postoperatively for splenic vein thrombosis over a period of 2 years, followed by withdrawal. An enhanced abdominal computed tomography (CT) scan showed no portal vein thrombosis 1 year prior to vaccination (Figure 4A,B), and Doppler ultrasonography revealed good portal vein flow along with stable liver function (aspartate aminotransferase [AST] 22 U/L, alanine aminotransferase [ALT] 17 U/L) 3 months before vaccination. The recipient experienced abdominal pain 2 weeks after the third vaccine dose (Moderna mRNA‐1273). The portal, superior, and inferior mesenteric veins were completely obstructed by thrombosis, as evidenced by an enhanced CT scan at our outpatient clinic (Figure 4C,D). Blood examination revealed liver dysfunction (AST 31 U/L, ALT 28 U/L, total bilirubin 1.98 mg/dL, prothrombin time international ratio [PT‐INR] 1.18). Hemolytic and anticoagulant reagents were administered systemically but were not effective. Case 2 was a 34‐year‐old female who had undergone living donor LT for fulminant hepatic failure using a left hemi‐liver graft donated by her father 19 years previously. One year prior to this study, she underwent splenectomy for hypersplenism following liver‐graft dysfunction due to chronic rejection. The portal vein was patent without any thrombosis 1 year before vaccination (Figure 4E) and showed good hepatopetal flow by Doppler ultrasonography 1 month before vaccination, along with slightly elevated liver enzyme levels and a normal coagulation test (AST 59 U/L, ALT 36 U/L, total bilirubin 1.36 mg/dL, PT‐INR 1.06). She experienced general fatigue with headache and nausea 2 weeks after the fourth vaccine dose (Pfizer‐BioNTech BNT162b2). She required emergency hospital admission because of pain in the hypochondrium and liver dysfunction (AST 216 U/L, ALT 108 U/L, total bilirubin 1.9 mg/dL). Using an enhanced CT scan, a partial thrombus in the intrahepatic portal vein was revealed (Figure 4F), and the thrombus size was reduced by administering anti‐thrombin‐III reagent.

## 4 DISCUSSION

The mRNA‐based vaccine against SARS‐CoV‐2 provided immunity to 89.1% and 100% of LT recipients after receiving the second and third doses, respectively. In addition, the titer was comparable to that of the healthy controls after the third dose. The humoral response rate in LT recipients post‐vaccination varied from 47.5% to 72%.^3^, ^4^, ^11^ Comparing Ab titers with previous reports is meaningless because there are many differences, including in the methods for measuring Ab titers and sample timing.^12^ Lower positive serology values and lower Ab titers have been reported in LT and kidney transplant recipients after the second vaccine dose, compared to those in healthy people.^3^, ^13^ Here, positive humoral response rates after the second and third doses were higher than those in previous reports, and the Ab titers after the third dose were comparable to those of healthy controls.

Immunosuppression may have a negative impact on vaccine immunization.^14^ Although tacrolimus,^15^ MMF,^16^ and steroids^3^ were reported to inhibit the SARS‐CoV‐2 vaccine immunization of organ recipients, these reagents or multiple immunosuppression regimens that include them were not associated with the humoral response of anti‐S Ab after the second vaccine dose in this study. A younger age (<47 years) at LT and longer duration between LT and vaccination (>2 years) were independent predictive factors for positive humoral response. Older age and shorter interval after LT were risk factors associated with non‐response to vaccination in LT recipients.^17^ The majority of recipients here had a longer duration between LT and the first vaccination, with an average interval of 15.2 years. They were already on very small doses of immunosuppressants and possibly similar to healthy people in terms of immunity; therefore, the positive humoral response rate might have been higher here than in previous reports.

Here, ~10% of recipients converted from negative‐ to positive‐Ab status after the third dose. It is unclear how much Ab titer is needed to prevent SARS‐CoV‐2 infection. In addition, the infection‐preventing effects of Abs differ depending on the viral strain, and different cut‐off values have been set for judging negatives and positives of between 0.8 and 50 AU/mL.^3^, ^9^, ^12^, ^15^ The Ab neutralization level to protect against symptomatic infection is 20.2%, which corresponds to 54 IU/mL of titer.^18^ Here, the cut‐off value was set at 1.0 AU/mL, according to the manufacturer's protocol. The Ab titers increased each time LT recipients received vaccinations, indicating the effect of multiple vaccinations. Booster vaccinations have been reported to increase neutralizing Ab titers in healthy people.^19^

Here, anti‐S Ab titers decreased in most LT recipients after each vaccine dose; therefore, a booster vaccination is necessary to prevent infection. Ab titers linearly decreased after vaccination against SARS‐CoV‐2, with a decay rate of ~40% in each passing month.^10^ Therefore, we calculated the decay rate by dividing the difference in Ab titers measured between two different points by the number of days elapsed and found they were comparable between LT recipients and healthy individuals. These results show it is safe for LT recipients to receive booster vaccines at similar intervals as healthy individuals. Infection with SARS‐CoV‐2 based on the detection of anti‐N Ab positivity occurred in a few LT recipients (4.0%) here, indicating that vaccinated LT recipients have low rates of COVID‐19 infection.

Portal vein thrombosis after vaccination was noted in two cases (1.6%). Both recipients had a predisposition to thrombosis, including a history of splenic vein thrombosis and graft dysfunction. Vaccination‐induced thromboembolic events have been reported in over 1000 cases and may occur in patients with a pre‐existing hypercoagulable state.^20^ Thrombosis mainly occurs after adenoviral vector‐based vaccination,^21^ but it is unclear whether mRNA‐based vaccination causes thrombosis.^22^ A SARS‐CoV‐2 vaccine‐associated portal vein thrombosis has been reported in only one case among a population of more than 130 000 vaccinated persons,^21^ although there is no evidence indicating which sites are more common to vaccine‐associated thrombosis.^23^ Thrombolytic and anticoagulant therapies have shown limited efficacy. For LT recipients with a predisposition to thrombosis, other prophylaxis options, such as monoclonal Ab therapy, should be considered as an alternative to vaccines.

The limitations of this study are that a small cohort was used (100 subjects from two hospitals) and that the timing of measuring Ab titers varied because samples were collected during outpatient visits. In addition, the characteristics of the small number of healthy clinical workers used as controls were dissimilar to those of LT recipients, and their samples were obtained only around the third vaccine dose. A larger prospective study is recommended, but new studies will be difficult as many people have already been vaccinated several times. However, this study is useful in proving the effectiveness of new vaccine formulations. While humoral immunity is important, T‐cell and innate immunity also play crucial roles in preventing viral infections. However, this study lacks data on T‐cell function and innate immunity. It would be beneficial to evaluate these factors in future research.

## 5 CONCLUSION

LT recipients can acquire immunity similar to that of healthy individuals using the SARS‐CoV‐2 vaccine. The Ab decay rate is the same as that in healthy people, and booster vaccinations should be performed in the same way. Based on our results, we recommend vaccination against SARS‐CoV‐2 in LT recipients; however, vaccination‐associated portal vein thrombosis warrants caution, especially in patients with a predisposition to thrombosis.

## AUTHOR CONTRIBUTIONS

AM was a major contributor in analyzing the data, writing the manuscript, and producing the figures and tables. MM conceived this study and YO, AS, KK, YM, TN, and KY provided suggestions. YO and YM also participated in obtaining informed consent from patients. YS was a major contributor in organizing this study and in revising the manuscript. All authors have read and approved the final manuscript.

## FUNDING INFORMATION

This research received no specific grants from any funding agency in the public, commercial, or not‐for‐profit sectors.

## CONFLICT OF INTEREST STATEMENT

The authors declare no conflicts of interest for this article.

## ETHICAL APPROVAL

The study was conducted in accordance with the Declaration of Helsinki and the protocol was approved by the Ethics Committee of Shinshu University (registration number: 5265). Written informed consent was obtained from all the patients and/or their families before inclusion in the study.
